# Dataset of 2-(2-(4-aryloxybenzylidene) hydrazinyl) benzothiazole derivatives for GQSAR of antitubercular agents

**DOI:** 10.1016/j.dib.2017.08.006

**Published:** 2017-08-09

**Authors:** Amit S. Tapkir, Sohan S. Chitlange, Ritesh P. Bhole

**Affiliations:** a Dr. D. Y. Patil Vidya Pratishthan Society's Dr. D.Y. Patil Institute of Pharmaceutical Sciences & Research, Pimpri, Pune 411018, Maharashtra, India; b Progressive Education Society's, Modern College of Pharmacy, Sector 21, Yamunanagar, Nigdi, Pune 411044, Maharashtra, India

**Keywords:** Antitubercular, Quantitative structure-activity relationship, GQSAR, Benzothiazole

## Abstract

Fragment based Quantitative structure activity relationship (QSAR) analysis on reported 25 2-(2-(4-aryloxybenzylidene) hydrazinyl) benzothiazole dataset as antitubercular agents were carried out. Molecules in the current dataset were fragmented into six fragments (R1, R2, R3, R4, R5, R6).Group based QSAR Models were derived using Multiple linear regression (MLR) analysis and selected on the basis of various statistical parameters. Dataset of benzothiazole reveled importance of presence of halogen atoms on is essential requirement. The generated models will provide structural requirements of benzothiazole derivatives which can be used to design and develop potent antitubercular derivatives.

**Specifications Table**

**Table:** 

Subject area	Computational and Insilico Chemistry
More specific subject area	Group Quantitative Structure-Activity Relationship(QSAR)
Type of data	Equation, Tables,Graphs
How data was acquired	Group based QSAR modelling
Data format	Analysis
Experimental factors	Multiple linear regression GQSAR models for predicting the inhibitory potential of benzothiazole dataset were created. 17 molecules were utilized as training dataset and 8 molecules utilized as test dataset.
Experimental features	Fragment descriptors and pMIC values were utilized in GQSAR analysis via stepwise variable selection method using dataset of 25 molecules.
Data source location	Pharmaceutical chemistry of Laboratory of Progressive Education Society's, Modern College of Pharmacy, Sector 21, Yamunanagar, Nigdi, Pune 411044, Maharashtra, India
Data accessibility	The data is with this article

**Value of the data**

• Tuberculosis is one of most lethal disease in the current decade; development of potent antitubercular compounds is need of time.
• GQSAR modelling data was developed for predicting structural properties of benzothiazole dataset which are infusing antitubercular activity.
• The GQSAR models generated will be utilized to screen various heterocyclic datasets for antitubercular potency, which will lead to development of novel antitubercular compounds.

## 1 Data

The data shown here regarding a GQSAR equation development that is used to predict contribution of substituents towards antitubercular potential of benzothiazole dataset.

## 2 Experimental design, materials and methods

### 2.1 Data set preparation

Molecular data set for current study were taken from literature reported by Telvekar et al. [1]. All the 24 structures of benzothiazole derivatives were drawn using 2D builder module of Vlife MDS 4.3. These 2D structures were converted into 3D via using V life engine platform. Geometry and structures of 3D molecules were optimized via energy minimization process using Merck molecular force field (MMFF) and Gasteiger charges. A common template which is a representative of the entire molecules under study was prepared with the presence of a dummy atom (X) at the substitution site.

### 2.2 Calculation of descriptors

**Fig. 1:**
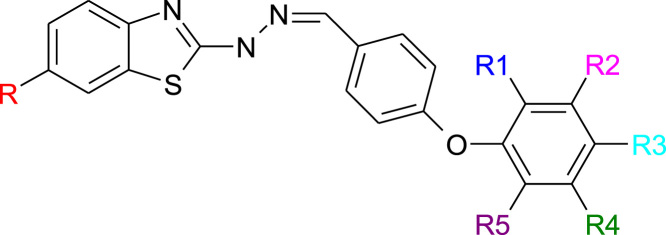
Molecular Template Utilized for Fragmentation pattern.

The common chemical structure as shown in Fig. 1 was utilized for development of GQSAR model. The molecules in the data set were fragmented in six different fragments (R-R6). The fragmented molecules were incorporated into the QSAR module of V life MDS for calculation of molecular descriptors. Molecular descriptors are nothing but the numerical values which represents physical and chemical information of the molecules. In GQSAR studies descriptors are representation of the physical and chemical behavior of substituents present.

### 2.3 Data selection and building G-QSAR model [2], [3], [4], [5]

Generated dataset of 25 benzothiazole derivatives were randomly divided into training set and test set 17 and 8 molecules respectively. Random distribution of training and test set will results into uniform distribution of biological activity across the molecules under study. Multiple linear regression analysis was utilized for development GQSAR models, with number of dependent variable limited to not more than 3 per model (Table 1).

**Table 1:** Table Showing Molecules under Study.

**Mole. No**	**R**	**R**_**1**_	**R**_**2**_	**R**_**3**_	**R**_**4**_	**R**_**5**_
1.	H	H	H	H	H	H
2.	H	Cl	H	H	H	H
3.	H	H	H	Cl	H	H
4.	H	Cl	H	Cl	H	H
5.	H	H	CH_3_	Cl	H	H
6.	Cl	H	H	H	H	H
7.	Cl	Cl	H	H	H	H
8.	Cl	H	H	Cl	H	H
9.	Cl	Cl	H	Cl	H	H
10.	Cl	H	CH_3_	Cl	H	H
11.	CH_3_	H	H	H	H	H
12.	CH_3_	Cl	H	H	H	H
13.	CH_3_	H	H	Cl	H	H
14.	CH_3_	Cl	H	Cl	H	H
15.	CH_3_	H	CH_3_	Cl	H	H
16.	OCH_3_	H	H	H	H	H
17.	OCH_3_	Cl	H	H	H	H
18.	OCH_3_	H	H	Cl	H	H
19.	OCH_3_	Cl	H	Cl	H	H
20.	OCH_3_	H	CH_3_	Cl	H	H
21.	NO_2_	H	H	H	H	H
22.	NO_2_	Cl	H	H	H	H
23.	NO_2_	H	H	Cl	H	H
24.	NO_2_	Cl	H	Cl	H	H
25.	NO_2_	H	CH_3_	Cl	H	H

### 2.4 Validation of the developed G-QSAR model [6], [7], [8], [9], [10]

Validation is a critical step in the QSAR model development. Validation methods are required for establishing predictability of QSAR model on unseen data and for determination of complexity of QSAR model which is justified by the data under study. Number of methods like the methods of least squares fit (R2), cross validation (Q2), adjusted R2 (R2adj), chi-squared test (χ2), root mean squared error (RMSE), bootstrapping and scrambling (Y-Randomization) are reported for internal validation of QSAR models. Observed activity of molecules in dataset was expressed in MIC(μg/ml) and converted into pMIC for QSAR analysis. All the molecules in the dataset are having activity (MIC) in the range 1.5–29.00 μg/ml.

### 2.5 QSAR analysis

**Fig. 2:**
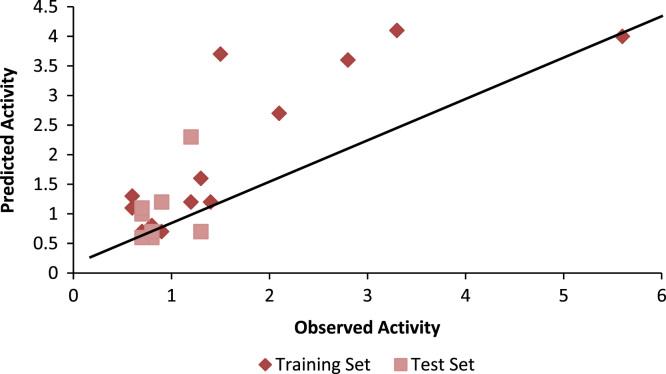
Figure Showing Correlation Plot for Selected GQSAR model having r^2^ 0.88.

Congeneric nature of the dataset is basis prerequisite for any QSAR analysis. Fragment based QSAR is recent methodology were complex structures can be analyzed. 30 different G-QSAR models were generated and best one of them are selected on basis of the statistical values like r^2^, q^2^, pred_r^2^, F-test and standard error. The predicted activity data via QSAR models was in accordance with the observed biological activity with small variations which were clearly identified in the correlation plot of different model (Table 2 and Fig. 2). Selected model is given by.

**Table 2:** Table showing observed and predicted activity of selected GQSAR model.

**Molecule. No**	**Observed Activity pMIC** **(μg/ml)**	**Predicted Activity pMIC** **(μg/ml)**	**Molecule No**	**Observed Activity pMIC** **(μg/ml)**	**Predicted Activity pMIC** **(μg/ml)**
1	2.1	2.7	14	0.9	1.2
2	0.9	0.7	15	0.8	0.8
3	1.2	1.2	16	0.8	0.7
4 #	2.3	1.2	17#	0.7	1.3
5#	1.2	0.9	18#	1.1	0.7
6	1.3	1.6	19	0.6	1.3
7	3.3	4.1	20	0.6	0.8
8	2.8	3.6	21#	0.6	0.7
9	5.6	4.0	22#	0.6	1.1
10	1.5	3.7	23	0.7	0.7
11#	1.0	0.7	24	0.7	1.1
12	1.4	1.2	25#	0.7	0.8
13	0.8	0.7			

# Test Set Molecules

pMIC: 0.0038+ 2.9110(±0.4296)R1-ChlorinesCount+ 5.5097(±2.0358) R2-MomInertiaX.

r^2^: 0.8845, q^2^: 0.6059, F test: 35.45.
