# Supplementary material for: Dataset of 2-(2-(4-aryloxybenzylidene) hydrazinyl) benzothiazole derivatives for GQSAR of antitubercular agents
Source: Data Brief. 2017 Aug 9;14:469–73. doi: 10.1016/j.dib.2017.08.006 (PMC5554989; doi:10.1016/j.dib.2017.08.006)
Supplement: Supplementary file 1 — Supplementary material [file mmc1.docx]

MANUSCRIPT TITLE:

**FRAGMENT BASED QSAR MODELLING ON DATASET OF 2-(2-(4-ARYLOXYBENZYLIDENE)HYDRAZINYL)BENZOTHIAZOLE DERIVATIVES AS ANTITUBERCULAR AGENTS**

Conflicts of interest Form

Authors has no Conflicts of interest.
